# Care‐Related Quality of Life of Caregivers for Patients With Low Vision and Blindness: A Cross‐Sectional Study

**DOI:** 10.1155/joph/4366827

**Published:** 2026-03-25

**Authors:** Yu Zhang, Liyan Yao, Xiaoxin Guo, Huiming Xiao, Wenmin Huang, Chunyan Yang

**Affiliations:** ^1^ State Key Laboratory of Ophthalmology, Zhongshan Ophthalmic Center, Sun Yat-sen University, Guangdong Provincial Key Laboratory of Ophthalmology and Visual Science, Guangdong Provincial Clinical Research Center for Ocular Diseases, Guangzhou, 510060, China, sysu.edu.cn

**Keywords:** caregiver, caregiving burden, low vision and blindness, quality of life, sense of coherence

## Abstract

**Purpose:**

Healthcare systems typically focus on patient care, often overlooking the substantial challenges faced by informal caregivers, particularly those caring for individuals with severe or chronic conditions. This study explores the relationships between care‐related quality of life (CarerQol), self‐rated caregiver burden, and sense of coherence (SOC), identifying potential predictors that influence CarerQol for caregivers of patients with low vision and blindness.

**Methods:**

This cross‐sectional, descriptive study assessed caregiving burden using the CarerQol instrument, which measures seven dimensions of subjective burden (CarerQol‐7D) and a happiness score (CarerQol‐VAS). Data on demographic variables, self‐rated caregiver burden, SOC, and the patients’ Barthel Index (BI) were collected. Univariate analyses were performed for each determinant, followed by multivariate regression analysis that included age, gender, education level, and variables with *p* < 0.10.

**Results:**

A total of 128 caregivers participated; 57.0% were male, and 64.0% were under 50 years of age. The mean CarerQol‐7D utility score was 77.15 ± 17.20, showing significant associations with the self‐rated caregiver burden (*r* = −0.596, *p* < 0.001) and SOC (*r* = 0.435, *p* < 0.001). The mean CarerQol‐VAS score was 6.49 ± 2.27, with significant correlations to self‐rated caregiver burden (*r* = −0.501, *p* < 0.001), SOC (*r* = 0.428, *p* < 0.001). Multivariate analysis revealed that higher education levels and family support were independently associated with higher CarerQol‐7D utility scores. Additionally, self‐rated caregiver burden was negatively correlated with both CarerQol‐7D utility and CarerQol‐VAS scores, while SOC was a positive predictor.

**Conclusion:**

This study highlights that higher educational levels, family support, lower caregiver burden, and a stronger SOC are key predictors of better CarerQol. Healthcare professional should focus on implementing effective interventions, such as providing healthcare education, encouraging family support, reducing caregiver burden, and strengthening the SOC, to enhance CarerQol.

## 1. Introduction

Low vision and blindness, often caused by conditions such as glaucoma, diabetic retinopathy, age‐related macular degeneration, cataracts, amblyopia, and strabismus [1–5], present significant challenges. A meta‐analysis estimates that 7.08 million people in the United States have a best‐corrected visual acuity (BCVA) of 20/60 or worse in their better‐seeing eye, with 5.46 million of these individuals over the age of 40. Among them, approximately 1.08 million are blind [6]. The prevalence of visual impairment increases with age, reaching 6.46% of individuals aged 65%–85% and 20.65% of those over 85, with females being more affected than males [7]. While healthcare systems primarily focus on patient care, informal caregivers often face substantial challenges, particularly when caring for individuals with severe or chronic conditions [8].

Informal caregiving, typically provided by family members or close friends, may involve assistance during medical visits, managing medications, wound care, and supporting other daily activities [9, 10]. This care is usually given voluntarily by nonprofessionals without compensation [10]. In addition to physical care, the emotional and social support from caregivers is essential for decision‐making and coping strategies [11, 12]. After diagnosis and treatment, individual with low vision and blindness often require continuous care and may experience disease progression [13]. Although many patients return home after initial treatment, they frequently need ongoing support for daily living activities [12].

Caregiving can be especially burdensome when individuals with low vision and blindness require specialized care [13]. While caregiving can foster personal growth for caregivers, it also brings considerable challenges [8]. The stress and anxiety associated with ongoing caregiving can negatively affect caregivers’ health, which may in turn impact the quality of care provided to the patient [14, 15]. Using standardized questionnaires to assess caregivers’ care‐related quality of life (CarerQol) can help identify potential financial, relational, or health‐related issues early [8]. Encouraging active caregiver involvement in the care process and improving communication among caregivers, patients, and healthcare providers can lead to better outcomes for both caregivers and patients [16].

Caregiver burden is a major source of stress, owing to the physical, psychological, emotional, social, and financial demands it places on caregivers [17]. As caregiver burden increases, so does the likelihood of negative health behaviors and increased healthcare utilization [18]. It is well‐established that caregiver burden negatively affects quality of life (Qol) [19] and overall well‐being [17, 20]. Furthermore, a high caregiver burden can compromise the quality of care provided to patients [20]. Caregivers of individuals with low vision and blindness, in particular, experience substantial burden, highlighting the need for further research on its effects on CarerQol in this population.

The sense of coherence (SOC), a fundamental concept in Antonovsky’s salutogenesis theory [21], refers to individuals’ belief that their lives are understandable, manageable, and meaningful. A strong SOC is linked to better stress management and health maintenance, as it enables individuals to effectively utilize available resources [22]. In contrast, caregivers with low SOC typically report poorer health, reduced Qol, and fewer social connections [22, 23]. Despite the known challenges of caregiving, research on the impact of caregiving for individuals with low vision and blindness remains limited. While studies on informal CarerQol provide some insights, there is a gap in understanding the specific needs of caregivers of individuals with low vision and blindness.

**FIGURE 1:**
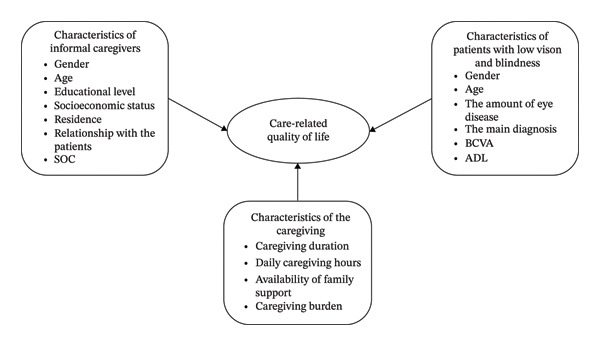
Conceptual framework of CarerQol and related variables.

Based on Antonovsky’s salutogenesis model [21], we hypothesize that individual characteristics of both patients and caregivers, caregiving burden, and SOC predict CarerQol among caregivers of individuals with low vision and blindness. Figure 1 illustrates the hypothesis framework of this study. This aim of this study is to explore relationships among CarerQol, caregiver burden, and SOC, with a focus on identifying factors that influence CarerQol. Ultimately, these findings may help identify areas where intervention could improve the lives of informal caregivers.

## 2. Methods

### 2.1. Study Design

We conducted a cross‐sectional investigation between July 2022 and September 2023.

### 2.2. Sampling and Inclusion Criteria

The informal caregiver was defined as the individual who spent the most time with the patient and played a key role in making decisions regarding their care, including family member, relatives, and friends [8, 12]. The inclusion criteria were as follows: informal caregivers of patients with BCVA of 20/60 or worse in their better‐seeing eye, including glaucoma, retinal diseases, and cornea diseases. We excluded patients with additional medical conditions requiring regular caregiving, in order to focus specifically on caregivers providing support due to the patient’s visual impairment. Exclusion criteria included conditions such as severe neurological disease, physical or mental disabilities, or any condition that impaired the patient’s ability to move independently. However, patients with manageable comorbidities, such as hypertension, early‐stage diabetes, hyperlipidemia, gout, or arthritis, were not excluded. Additionally, caregivers who received payment for caregiving and those who could not understand or complete the questionnaire were excluded.

The sample size was determined using the formula $n={\left(U_{\alpha /2}\sigma /\delta \right)}^{2}$, where *U*_*α*/2_ = 1.96, *σ* = 77.6, and *δ* = 17.4 which were based on the mean and standard deviation of CarerQol‐7D utility observed among the informal caregivers of people with dementia from eight European countries [24]. The initial estimate indicated a need for 77 participants; however, to account for a potential 10% nonresponse rate, the minimum sample was raised to 84. Additionally, the multiple linear regression requirement of 5–10 participants per independent variable [25], with 16 independent variables, suggested a range of 80–160 participants. Allowing for a potential 20% invalid response rate, at least 96 valid samples were needed. Ultimately, 128 participants were deemed sufficient for the study.

### 2.3. Data Collection

A face‐to‐face survey was conducted with caregivers caring for patients with low vision and blindness who were either receiving treatment in an inpatient unit or visit the outpatient clinic at a tertiary‐level ophthalmic specialty hospital in Guangzhou, Guangdong Province. Trained researchers, who had undergone comprehensive training in the study protocol, standardized survey procedures, and questionnaire management, conducted the data collection. They strictly adhered to inclusion and exclusion criteria for participants’ recruitment and ensured quality control throughout the process. In the formal survey, uniform instructions were provided to participants regarding the purpose of the survey and how to complete the questionnaire, and questionnaires were administered only after obtaining informed consent from each participant. Participants were informed that their responses would remain anonymous and confidential, and that all data would be used solely for research purposes. Questionnaires were self‐administered, with researchers available to clarify procedures if needed. On completion, researchers reviewed each questionnaire on site for completeness, and any missing information was promptly verified and supplemented through direct communication with the participants.

### 2.4. Ethical Considerations

Caregivers were provided with verbal information about the study’s purpose at the hospital. They were informed that participation was voluntary and that they could withdraw at any time without consequence. Anonymity was guaranteed for all participants. Informed consent was obtained from those who agreed to participate. The study was approved by the Ethics Committee of the Zhongshan Ophthalmic Center, Sun Yat‐sen University, under the approval number 2024LSPJ227.

### 2.5. Measurements

#### 2.5.1. Sociodemographic Characteristics

The demographic characteristics and disease‐related character of patients included their age, gender, number of eye conditions, and BCVA. Informal caregivers provided data on their own demographic details, including age, gender, educational level, household per capita monthly income (in CNY), place of residence, and their relationship to the patient. Additionally, caregiving time burden was assessed by evaluating the caregiving duration, daily caregiving hours, and the availability of family support.

#### 2.5.2. CarerQol

The impact of caregiving on the Qol of informal caregivers was assessed using the CarerQol instrument [26], which has been validated in multiple settings, including hospitals, long‐term care facilities, and primary care centers. The Chinese version of the CarerQol was introduced and culturally adapted by Zhang Yuehua [27], who demonstrated that the Dutch tariff weights developed by Hoefman et al. [28] showed strong agreement with directly summed dimension scores in a Chinese population. The CarerQol consists of two parts: the CarerQol‐7D and the CarerQol Visual Analog Scale (CarerQol‐VAS). The CarerQol‐7D measures caregiver burden across seven dimensions, with two positive aspects (care‐related fulfillment and social support) and five negative aspects (relationship issues with the care recipient, mental health concerns, financial insecurity, challenges with daily activities, and physical health issues). Each dimension is rated on a three‐level scale (no, some, or a lot of). Utility scores are derived using a tariff based on population preferences from the Dutch [28], allowing the generation of standardized scores ranging from 0 (*poorest caregiving situation*) to 100 (*best caregiving situation*). In the present study, the utility‐based CarerQol‐7D scores showed a strong correlation with directly summed dimension scores (*r* = 0.947, *p* < 0.001), supporting the applicability of the utility scoring approach in this sample. Although Cronbach’s *α* was 0.666, which was slightly below the conventional threshold, this is consistent with the multidimensional nature of the instrument. The CarerQol‐VAS provides an additional measure of general well‐being, specifically happiness, using a visual scale from 0 (*completely unhappy*) to 10 (*completely happy*).

#### 2.5.3. Self‐Rated Caregiver Burden

The Zarit Burden Interview (ZBI), developed by Zarit et al. in 1980 [29–31], measures multiple dimensions of caregiver burden, including psychological well‐being, health, social life, financial strain, and the caregiver’s relationship with the patient. This tool consists of 22 items, each scored on a five‐point Likert scale, with a total score range from 0 to 88. Individual items are rated from 0 (*never*) to 4 (*nearly always*), except for the final item on overall burden, which is rated from 0 (*not at all*) to 4 (*extreme burden*). The ZBI has demonstrated high reliability, with test–retest reliability of 0.71 and internal consistency of 0.92 in the original study. Cronbach’s *α* was 0.910 in our study population, demonstrating good reliability and validity.

#### 2.5.4. SOC

The SOC was assessed using the short version of the Orientation to Life Questionnaire [21], which includes 13 items and has shown a strong correlation with the longer original form. The SOC‐13 uses a seven‐point Likert scale, ranging from “*seldom or never*” [1] to “*very often or always*” [7]. Total scores range from 0 to 91, with higher scores indicating better coping abilities in stressful situations. The questionnaire covers three dimensions of SOC: Meaningfulness (four items, e.g., “How often do you feel that there’s little meaning in the things you do daily?”); Comprehensibility (five items, e.g., “Do you feel that you’re in an unfamiliar situation and don’t know what to do?”); Manageability (four items, e.g., “How often do you feel unsure you can keep things under control?”). Five negatively worded items are reverse‐coded to ensure that higher scores correspond to stronger SOC. The SOC‐13 has been proven an effective measurement in Chinese patients, with Cronbach’s *α* of 0.760 [32]. In the current study, Cronbach’s *α* for this scale was 0.737.

#### 2.5.5. Activity of Daily Living

The activity of daily living of patient was evaluated using the Barthel Index (BI), a tool developed by Mahoney et al. [33]. The BI assesses the level of dependency in 10 key daily activities: feeding, bathing, grooming, dressing, bowel and bladder control, toilet use, transferring, mobility, and stair climbing. Each activity is scored up to 15 points, with the total score ranging from 0 to 100. A score of 100 indicates complete independence, while lower scores reflect increasing levels of dependency on caregiving. In the present study, Cronbach’s *α* was 0.838, demonstrating good internal consistency.

### 2.6. Statistical Analysis

Descriptive statistics, including frequencies and proportions, were used to summarize the characteristics of both patients and informal caregivers. The normality of the data was assessed using the Kolmogorov–Smirnov and Shapiro–Wilk tests. Mean and standard deviation were calculated for the overall CarerQol, SOC, ZBI, and BI, while the distribution of responses for each CarerQol‐7D dimension was expressed as percentages.

Pearson correlation analysis was conducted to examine the relationships between CarerQol scores and SOC, ZBI, and BI. A *t*‐test or analysis of variance (ANOVA) was applied to compare CarerQol among caregivers with different characteristics. For the multivariable linear regression analysis, CarerQol‐7D utility, and CarerQol‐VAS scores served as dependent variables, with any variable having *p* < 0.10 included as an independent variable. Results were reported as beta coefficients, and model fit was evaluated using *R* ^2^ values. Statistical significance was set at a *p* < 0.05. All analyses were performed using SPSS, Version 25.0 (IBM Corporation, Armonk, NY, USA).

## 3. Results

### 3.1. Sociodemographic Characteristics

A total of 128 caregivers participated in the study. Table 1 presents the characteristics of patients with low vision and blindness. Among the patients, 51.6% (*n* = 66) were female, and 48.4% (*n* = 62) were male. Age group included ≤ 30 years old (*n* = 12; 9.4%), 31–50 years old (*n* = 32; 25.0%), and ≥ 51 years old (*n* = 84; 65.6%). Approximately 47.7% (*n* = 61) had one eye disease, while 52.4% (*n* = 67) had two or more eye diseases. Glaucoma accounted for 71.1% (*n* = 91) of low vision or blindness, and diabetic retinopathy accounted for 28.9% (*n* = 37). Out of the total, there were 64 patients (50%) with BCVA > 0.05 in both eyes, while 23 patients (18.0%) had at most one eye with BCVA ≤ 0.05, and 41 patients (32.0%) had BCVA ≤ 0.05 in both eyes.

**TABLE 1:** Characteristics of patients with low vision or blindness (*N* = 128).

Patients		*N* (%)/Mean ± SD
Gender	Male	62 (48.4)
	Female	66 (51.6)

Age (years)	≤ 30	12 (9.4)
	31–50	32 (25.0)
	≥ 51	84 (65.6)

The amount of eye disease	≤ 1	61 (47.7)
	≥ 2	67 (52.4)

The type of eye disease	Diabetic retinopathy	37 (28.9)
	Glaucoma	91 (71.1)

BCVA	0.05 < both eyes ≤ 0.3	64 (50.0)
	One eye ≤ 0.05	23 (18.0)
	Both eyes ≤ 0.05	41 (32.0)

BI		76.41 ± 15.08

Abbreviations: BCVA, best‐corrected visual acuity; BI, Barthel Index; SD, standard deviation.

The demographic characteristics and time burden of caregiving of caregivers were shown in Table 2. The majority of informal caregivers in this study were male (*n* = 74, 57.8%). Caregivers were categorized into three age groups: 20 (15.6%) were aged 30 years or younger, 62 (48.4%) were between 31 and 50 years old, and 46 (36.0%) were 51 years or older. Most caregivers were either the patients’ spouses (*n* = 54, 42.2%) or children (*n* = 50, 39.1%). Nearly half of the caregivers (*n* = 57, 44.5%) had an educational level of middle school or lower. Regarding household per capita monthly income, 74.2% (*n* = 95) reported earnings of 8000 CNY or less. Additionally, a significant proportion (*n* = 81, 63.3%) resided in rural areas.

**TABLE 2:** Characteristics of caregivers for patients with low vision or blindness (*N* = 128).

Caregiver		*N* (%)
Gender	Male	74 (57.8)
	Female	54 (42.2)

Age	≤ 30	20 (15.6)
	31–50	62 (48.4)
	≥ 51	46 (36.0)

Educational level	Junior school or lower	57 (44.5)
	Senior and technical school	31 (24.2)
	College or above	40 (31.3)

Household per capita monthly income	≤ 8000	95 (74.2)
	> 8000	33 (25.8)

Residence	Urban	47 (36.7)
	Rural	81 (63.3)

Relationship with the patient	Spouse	54 (42.2)
	Child	50 (39.1)
	Other	24 (18.7)

Caregiving duration (years)	≤ 1	88 (68.8)
	1–2	22 (17.2)
	≥ 2	18 (14.1)

Daily caregiving hours, *n* = 125	≤ 8	87 (69.6)
	9–16	19 (15.2)
	≥ 17	19 (15.2)

Availability of family support, *N* (%)	No	53 (41.4)
	Yes	75 (58.6)

Of the caregivers surveyed, 18 (14.1%) have been providing care for more than two years, 22 (17.2%) for one to two years, and 88 (68.8%) for one year or less. With regard to the daily caregiving time, 19 (15.2%) reported providing care for over 17 h per day, 19 (15.2%) for 9–16 h, and 87 (69.6%) for 8 h or fewer. More than half of the caregivers (*n* = 75; 58.6%) had family members providing help.

### 3.2. Primary Outcome

**FIGURE 2:**
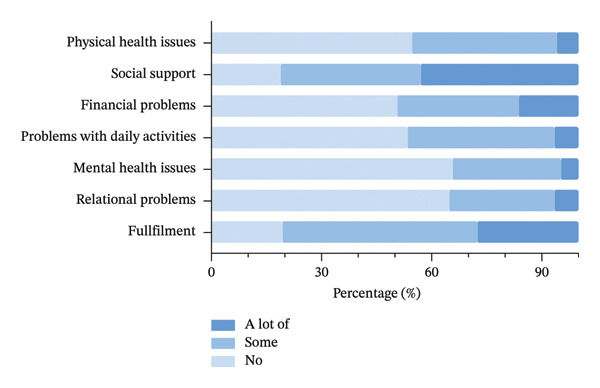
Distribution of responses to the CarerQol‐7D in caregivers of patients with low vision and blindness.

Utilizing the Dutch tariff for the CarerQol‐7D, the mean CarerQol‐7D utility score was 77.15 ± 17.20. Figure 2 illustrates the distribution of responses across the CarerQol survey dimensions. Among the positive dimensions, most caregivers reported experiencing some or a lot of fulfillment (80.5%) and social support (81.3%). The mean CarerQol‐VAS score was 6.49 ± 2.27, reflecting caregivers’ overall well‐being, specifically happiness. In addition, the mean scores for the ZBI and SOC were 22.46 ± 14.71 and 61.59 ± 8.61, respectively. Detailed results are presented in Table 3.

**TABLE 3:** Scores of primary outcome measures in our study (*N* = 128).

Variables	Mean ± SD
CareQol‐7D utility score	77.15 ± 17.20
CareQol VAS score	6.49 ± 2.27
ZBI	22.46 ± 14.71
SOC	61.59 ± 8.61

*Note:* CareQol, care‐related quality of life.

Abbreviations: SD, standard deviation; SOC, sense of coherence; VAS, visual analog scale; ZBI, Zarit Burden Interview.

### 3.3. Correlations Between Caregivers’ CarerQol Scores, ZBI, SOC, and Patients’ BI

A significant negative correlation was found between the CarerQol‐7D utility scores and ZBI (*r* = −0.596, *p* < 0.001), and a positive correlation with the SOC (*r* = 0.435, *p* < 0.001). However, the CarerQol‐7D utility scores did not show a significant correlation with patients’ BI (*r* = 0.133, *p* = 0.134). The CarerQol‐VAS score was significantly negatively correlated with the ZBI (*r* = −0.501, *p* < 0.001) and positively correlated with the SOC (*r* = 0.428, *p* < 0.001). Additionally, a positive relationship was observed between the CarerQol‐VAS score and patients’ BI (*r* = 0.249, *p* = 0.005). A medium positive correlation was also found between CarerQol‐7D utility and CarerQol‐VAS scores (*r* = 0.612, *p* < 0.001). Detailed results are presented in Table 4.

**TABLE 4:** Pearson correlation coefficients between the scales (*N* = 128).

	**CarerQol-7D utility score**	**CarerQol-VAS score**	**ZBI**	**SOC**	**BI**

Informal caregivers					
CarerQol‐7D utility score		0.612 (*p* < 0.001)^∗^	−0.596 (*p* < 0.001)^∗^	0.435 (*p* < 0.001)^∗^	0.133 (*p* = 0.134)
CarerQol VAS score	0.612 (*p* < 0.001)^∗^		−0.501 (*p* < 0.001)^∗^	0.428 (*p* < 0.001)^∗^	0.249 (*p* = 0.005)^∗^
ZBI	−0.596 (*p* < 0.001)^∗^	−0.501 (*p* < 0.001)^∗^		−0.461 (*p* < 0.001)^∗^	−0.370 (*p* < 0.001)^∗^
SOC	0.435 (*p* < 0.001)^∗^	0.428 (*p* < 0.001)^∗^	−0.461 (*p* < 0.001)^∗^		0.086 (*p* = 0.336)

Patients					
BI	0.133 (*p* = 0.134)	0.249 (*p* = 0.005)^∗^	−0.370 (*p* < 0.001)^∗^	0.086 (*p* = 0.336)	

*Note:* CarerQol, care‐related quality of life.

Abbreviations: BI, Barthel Index; SOC, sense of coherence; VAS, visual analog scale; ZBI, Zarit Burden Interview.

^∗^Significant at the *p* < 0.05 level.

### 3.4. Univariate and Multivariable Regression Analyses

Stepwise multiple linear regression analysis was conducted to identify variables significantly associated with the CarerQol‐7D utility and CarerQol‐VAS scores. Variables with *p* < 0.10 were included in the models. For CarerQol‐7D utility scores, the analysis considered patient gender, BCVA, caregiver educational level, household per capita monthly income, caregiver residence, caregiving duration, availability of family support, ZBI, and SOC score. Similarly, factors analyzed for CarerQol‐VAS scores included patient gender, BCVA, patient’ BI, caregiver residence, educational level, daily caregiving hours, availability of family support, ZBI, and SOC score. Categorical demographic variables were dummy‐coded for analysis. Full details are provided in Table 5.

**Table 5:** Univariate regression analyses for the CarerQol‐7D utility and CarerQol‐VAS score (*N* = 128).

		*n*	CarerQol‐7D utility score	*t*/*F*	*p*	CarerQol‐ VAS score	*t/F*	*p*
*Patients*								
Gender	Male	62	73.59 ± 20.23	−2.279	0.025^∗^	6.10 ± 2.42	−1.930	0.056
	Female	66	80.50 ± 13.06			6.86 ± 2.07		

Age (years)	≤ 30	12	77.18 ± 24.83	0.956	0.387	6.67 ± 2.16	0.557	0.574
	31–50	32	73.57 ± 16.73			6.13 ± 2.24		
	≥ 50	84	78.52 ± 16.11			6.61 ± 2.31		

The amount of eye disease	≤ 1	61	74.96 ± 17.97	−1.384	0.169	6.19 ± 2.53	−1.450	0.150
	≥ 2	67	79.16 ± 16.35			6.77 ± 1.98		

The type of eye disease	Diabetic retinopathy	37	76.60 ± 13.48	−0.233	0.816	6.50 ± 2.19	0.025	0.980
	Glaucoma	91	77.38 ± 18.57			6.49 ± 2.31		

BCVA	0.05 < Both eyes ≤ 0.3	64	77.29 ± 15.49 ①	2.307	0.104 ②③ (*p* = 0.034^∗^)	6.84 ± 2.13 ①	2.043	0.134 ①③(*p* = 0.046^∗^)
	One eye ≤ 0.05	23	83.11 ± 13.16 ②			6.54 ± 2.43 ②		
	Both eyes ≤ 0.05	41	73.59 ± 20.81 ③			5.93 ± 2.27 ③		

*Informal caregivers*								
Gender	Male	74	78.59 ± 16.77	1.109	0.270	6.62 ± 2.25	0.754	0.453
	Female	54	75.18 ± 17.75			6.32 ± 2.30		

Age (years)	≤ 30	20	77.29 ± 18.53	0.055	0.946	5.70 ± 2.58	3.147	0.046^∗^
	31–50	62	77.60 ± 16.81			6.98 ± 2.13		
	≥ 50	46	76.49 ± 17.51			6.19 ± 2.21		

Educational level	Junior school or lower	57	70.22 ± 18.92	9.692	< 0.001^∗^	5.78 ± 2.43	5.406	0.006^∗^
	Senior and technical school	31	81.30 ± 14.29			7.02 ± 2.01		
	College or above	40	83.82 ± 12.78			7.10 ± 1.96		

Household per capita monthly income (CNY)	≤ 8000	95	75.56 ± 18.28	−1.795	0.075	6.35 ± 2.41	−1.181	0.240
	> 8000	33	81.75 ± 12.81			6.89 ± 1.80		

Residence	Rural	47	82.49 ± 13.57	2.740	0.007^∗^	7.13 ± 1.91	2.459	0.015^∗^
	Urban	81	74.06 ± 18.37			6.12 ± 2.39		

Caregiving duration (years)	≤ 1	88	79.06 ± 1.69	2.859	0.061	6.68 ± 0.24	2.065	0.131
	1–2	22	76.50 ± 3.59			6.55 ± 0.52		
	≥ 2	18	68.61 ± 5.13			5.50 ± 0.50		

Daily caregiving hours, *n* = 125	≤ 8	87	78.90 ± 1.76	1.641	0.198	6.91 ± 0.21	4.939	0.009^∗^
	9–16	19	74.04 ± 4.93			5.71 ± 0.54		
	≥ 17	19	71.86 ± 3.87			5.42 ± 0.74		

Availability of family support, *N* (%)	No	53	71.44 ± 18.07	−3.278	0.001^∗^	5.76 ± 2.51	−3.199	0.002^∗^
	Yes	75	81.19 ± 15.45			7.01 ± 1.94		

*Note:* CarerQol, care‐related quality of life.

Abbreviations: BCVA, best‐corrected visual acuity; SD, standard deviation; VAS, visual analog scale.

^∗^Significant at the *p* < 0.05 level.

As shown in Table 6, caregiver educational level, availability of family support, ZBI, and SOC accounted for 44.6% of the variance in CarerQol‐7D utility scores. Patient gender and caregiver residence were excluded from the final model. For CarerQol‐VAS scores, significant predictors included daily caregiving hours, availability of family support, ZBI, and SOC, which together explained 38.3% of the variance. Other variables, such as caregiver residence, and education level were excluded from this model.

**TABLE 6:** Multivariate linear regression coefficients for the CarerQol‐7D utility and CarerQol‐VAS score (*N* = 128).

	CarerQol‐7D utility score			CarerQol‐VAS score		
	*B*	SE	*p*	*B*	SE	*p*
Care recipient						
Gender	3.804	2.312	0.103	0.516	0.335	0.127
BCVA	0.568	1.305	0.664	−0.006	0.202	0.975
BI				0.013	0.013	0.324
Informal caregivers						
Residence	0.274	2.680	0.919	0.156	0.375	0.678
Educational level	4.018	1.459	0.007^∗^	0.185	0.170	0.278
Household per capita monthly income	−0.196	2.830	0.945			
Caregiving duration	−0.371	1.667	0.824			
Daily caregiving hours				−0.506	0.254	0.049^∗^
Availability of family support	5.308	2.459	0.033^∗^	0.927	0.366	0.013^∗^
ZBI	−0.482	0.097	< 0.001^∗^	−0.042	0.014	0.004^∗^
SOC	0.486	0.152	0.002^∗^	0.082	0.022	< 0.001^∗^
Adjust *R* ^2^			0.446			0.383

*Note: B* unstandardized regression coefficient; CarerQol, care‐related quality of life; *R* ^2^ variance explained.

Abbreviations: BCVA, best‐corrected visual acuity; BI, Barthel Index; SE, standard error; SOC, sense of coherence; VAS, visual analog scale; ZBI, Zarit Burden Interview.

^∗^Significant at the *p* < 0.05 level.

## 4. Discussion

While significant attention is often paid to patients with low vision and blindness, the needs of their caregivers are frequently overlooked in clinical practice. This study aimed to assess the CarerQol of informal caregivers for patients with low vision and blindness, using both CarerQol‐7D utility and VAS scores. Unlike traditional caregiver burden scales, the CarerQol instrument offers a more comprehensive evaluation by evaluating seven dimensions of caregiving burden, each assigned a weighted score.

Our results indicated that caregivers generally reported a moderate CarerQol, as reflected by a mean CarerQol‐7D utility score of 77.15 ± 17.20 and a mean CarerQol‐VAS score of 6.49 ± 2.27. Most patients in our sample experienced low vision or blindness due to glaucoma, the leading irreversible cause of blindness worldwide; however, no significant difference in CarerQol emerged between caregivers of glaucoma patients and those of diabetic retinopathy patients. These findings suggest that caregiving responsibilities remain broadly similar, regardless of the underlying eye condition. While patients with diabetic retinopathy may require additional diabetes management support, the heightened anxiety and depression commonly seen in glaucoma patients likewise increase caregivers’ overall burden. Notably, our CarerQol‐7D utility and VAS scores were comparable to those reported for dementia caregivers in eight European countries [24], but caregivers in this study appeared to bear a higher burden than those identified in certain geriatric caregiving research [8], and a lower burden compared with caregivers of stroke survivors [18]. These findings underscore the importance of directing special attention to caregivers of individuals with low vision and blindness, particularly as current policies encourage individuals with vision impairment to lead fulfilling lives at home. As the global population with low vision and blindness increases, it becomes increasingly critical to provide sufficient support for informal caregivers in managing these essential caregiving responsibilities.

Interestingly, caregiving duration and daily caregiving hours were not correlated with higher CarerQol‐7D utility scores. This suggests that spending more time on caregiving or having more experience does not necessarily improve CarerQol. A similar study involving caregivers of patients with disabilities found no significant correlation between caregiving duration and role preparedness [34], whereas in a study on caregivers of cancer patients, preparedness was significantly and negatively associated with caregiver burden [35]. In contrast, our study observed that caregivers with higher education levels had better CarerQol‐7D utility scores, suggesting they may be better equipped for caregiving. Additionally, family support was linked to higher CarerQol scores, likely due to the benefits of a strong caregiver–patient relationship. Over 80% of caregivers in this study were spouses or children, and 64.0% were under the age of 50. Many caregivers struggled to balance caregiving with work, social activities, and other responsibilities, underscoring the importance of providing both physical and psychological respite. Of particular note, those who provided care for 9–16 h per day reported the highest well‐being levels, suggesting that this schedule may facilitate balancing caregiving with other life demands. These findings emphasize the value of interventions focused on disease management, patient care, and emotional support to enhance CarerQol [36–40].

Because we used the CarerQol instrument instead of traditional burden scales, direct comparisons with other studies are limited. Typically, a patient’s functional ability is closely related to caregiver’s burden, as more severely impaired patients often require more extensive care [41, 42]. In our sample, however, the ability to perform daily activities did not significantly correlate with CarerQol. One possible explanation is that most care in this study took place at home, whereas some research studies on caregiver burden often focus on inpatient settings, such as poststroke units [43]. A study of poststroke caregivers found that older caregiver age and patient disability were independent predictors of poorer health‐related quality of life (HRQol) [43]. Conversely, caregiver age was not a significant determinant of CarerQol in our investigation, aligning with the findings by van Dam et al. [8]. This discrepancy may reflect differences in outcome measures: While HRQol is more likely to be influenced by caregiver age and health status, CarerQol may be more strongly shaped by factors such as general health, happiness, and cognitive impairment.

In line with previous research, our study found a strong positive correlation between caregivers’ SOC and CarerQol scores. Caregivers who reported higher SOC also demonstrated better CarerQol, aligning with a Japanese study that emphasized the benefits of reinforcing SOC to enhance QoL [44]. Similarly, work conducted in both Western and Eastern countries has shown that caregivers with stronger SOC experienced less burden [45–48], possibly because they view caregiving as a meaningful, manageable, and comprehensible task. These outcomes underline the need to nurture SOC among caregivers to reduce burden and improve CarerQol. Healthcare professionals could achieve this by offering educational resources, medical advice, symptom relief, and psychological support, ultimately enhancing caregivers’ SOC and well‐being [49].

Consistent with Antonovsky’s salutogenesis model [21], our findings were interpreted within a theoretically coherent framework in which SOC represented a relatively stable orientation shaping individuals’ responses to stress. Within this framework, general resistance resources—such as higher educational level and family support—were found to strengthen SOC, thereby facilitating more effective appraisal and management of caregiving demands. Caregivers with stronger SOC consequently tended to report lower caregiver burden and better CarerQol. This theoretical positioning enhanced the plausibility of the associations observed in our cross‐sectional data and suggested that SOC and caregiving resources were more likely antecedents of CarerQol. Support for this pathway has also been provided by longitudinal evidence, indicating that caregiver burden changed meaningfully over time and prospectively predicted Qol in oncology cohorts [50]. Moreover, recent studies have identified SOC as a key psychosocial resource, demonstrating that higher SOC predicted more favorable caregiving burden trajectories and may act as a mediator in explanatory models of CarerQol [51, 52]. Together, these theoretical and longitudinal findings strengthened the interpretability of our results and suggested that interventions aimed at both reducing caregiver burden and enhancing SOC may represent effective strategies for improving CarerQol.

Despite these valuable insights, our study had several limitations. First, owing to its cross‐sectional design, the associations among variables could not be interpreted causally. Although our interpretations were guided by Antonovsky’s salutogenesis model and supported by longitudinal evidence from previous studies, reciprocal relationships could not be excluded. For instance, SOC may have mediated the relationship between caregiving burden and CarerQol. A larger, multicenter sample would enable a more robust analysis of the interaction mechanisms among caregiving burden, SOC, and CarerQol. Additionally, a longitudinal design would be necessary to clarify the directionality and dynamic evolution of these relationships. Second, because some data were collected through self‐reported questions, both recall and comprehension biases may have influenced the accuracy of the information. For example, caregivers may have underestimated or overestimated caregiving hours due to difficulty recalling or social desirability. Finally, we did not measure patients with severe comorbidities such as neurological disease and physical or mental disabilities, which may have influenced caregiving burden and limited the interpretation of some findings. Future research should consider additional factors, including sleep quality, depression, and disease awareness, to further enrich our understanding of caregiving experiences in low vision and blindness.

## 5. Conclusion

In conclusion, this study is the first to evaluate the CarerQol instrument specifically for caregivers of visually impaired patients. Most caregivers reported moderate CarerQol. Higher educational levels, family support, lower caregiver burden, and a stronger SOC were key predictors of better CarerQol. Healthcare professional should focus on implementing effective interventions, such as providing healthcare education, encouraging family support, reducing caregiver burden, and strengthening the SOC, to enhance CarerQol.

## Author Contributions

Yu Zhang contributed to the conceptualization, investigation, writing–original draft, methodology, writing–review and editing, visualization, project administration, data curation, resources, and software. Liyan Yao and Xiaoxin Guo contributed to the validation, software, formal analysis, and data curation. Huiming Xiao contributed to the conceptualization, investigation, methodology, data curation, and project administration. Wenmin Huang and Chunyan Yang contributed to the project administration, supervision, methodology, conceptualization, investigation, writing–review and editing, resources, and validation.

## Funding

This work was supported by the Nursing Innovation and Development Research Project of the Lingnan Nightingale Nursing Research Institute and Nursing Association, Guangdong Province (GDHLYJYB202403, gdhlxueh2019zl036), Research Project on Party Building Theory and Practice at Zhongshan Ophthalmic Center in 2024, Medical Science and Technology Foundation of Guangdong Province (A2021088, A2019365), and Nurturing funds for nursing young talents of Sun Yat‐sen University (N2018Y09).

## Conflicts of Interest

The authors declare no conflicts of interest.

## Data Availability

The data that support the findings of this study are available from the corresponding authors upon reasonable request.
